# Immune Mediators Profiles in the Aqueous Humor of Patients with Simple Diabetic Retinopathy

**DOI:** 10.3390/jcm12216931

**Published:** 2023-11-05

**Authors:** Naoyuki Yamakawa, Hiroyuki Komatsu, Yoshihiko Usui, Kinya Tsubota, Yoshihiro Wakabayashi, Hiroshi Goto

**Affiliations:** Department of Ophthalmology, Tokyo Medical University, 6-7-1 Nishi-shinjuku, Shinjuku-ku, Tokyo 160-0023, Japan; yamakawa@tokyo-med.ac.jp (N.Y.); v06058@gmail.com (H.K.); tsubnkin@hotmail.co.jp (K.T.); wbaki@tokyo-med.ac.jp (Y.W.); goto1115@tokyo-med.ac.jp (H.G.)

**Keywords:** diabetic retinopathy, simple diabetic retinopathy, immune mediator, cytokine, chemokine, growth factor

## Abstract

Various immune mediators identified to date are associated with the development of advanced forms of diabetic retinopathy (DR), such as proliferative DR and diabetic macular edema, although the exact pathophysiological mechanisms of early stages of DR such as simple DR remain unclear. We determined the immune mediator profile in the aqueous humor of eyes with simple DR. Fifteen eyes of fifteen patients with simple DR were studied. Twenty-two eyes of twenty-two patients with cataracts and no DR served as controls. Undiluted aqueous humor samples were collected, and a cytometric bead array was used to determine the aqueous humor concentrations of 32 immune mediators comprising 13 interleukins (IL), interferon-γ, interferon-γ-inducible protein-10 (IP-10), monocyte chemoattractant protein-1, macrophage inflammatory protein (MIP)-1α, MIP-1β, regulated on activation, normal T cell expressed and secreted (RANTES), monokine induced by interferon-γ, basic fibroblast growth factor (bFGF), Fas ligand, granzyme A, granzyme B, interferon-inducible T-cell alpha chemoattractant (ITAC), fractalkine, granulocyte macrophage colony-stimulating factor, granulocyte colony-stimulating factor (G-CSF), vascular endothelial growth factor (VEGF), angiogenin, tumor necrosis factor-α, and CD40 ligand. Among the 32 immune mediators, 10 immune mediators, including bFGF, CD40 ligand, fractalkine, G-CSF, IL-6, IL-8, MIP-α, MIP-1β, and VEGF, showed significantly higher aqueous humor concentrations and the Fas ligand had significantly lower concentration (*p* < 0.05) in eyes with simple DR compared with control eyes. Of these 10 cytokines with significant concentration alteration, protein–protein interaction analysis revealed that 8 established an intricate interaction network. Various immune mediators may contribute to the pathogenesis of simple DR. Attention should be given to the concentrations of immune mediators in ocular fluids even in simple DR. Large-scale studies are warranted to assess whether altered aqueous humor concentrations of these 10 immune mediators are associated with an increased risk of progression to advanced stages of DR.

## 1. Introduction

Diabetic retinopathy (DR) is the major cause of blindness in the older population. Currently, 284.6 million people worldwide are estimated to have diabetes mellitus; approximately one third of those with diabetes are at risk of developing DR to some extent, and approximately one third of those with DR may advance to the vision-threatening stage [1]. Two major advanced forms of DR, diabetic macular edema and proliferative DR (PDR), develop from pre-retinal neovascularization (abnormal growth of new blood vessels) that causes the majority of diabetes-related severe visual impairment. Microaneurysms, retinal hemorrhages, and hard or soft exudates occur early in DR development, and characterize simple DR or non-proliferative DR. The progression of simple DR to subsequent stages like pre-PDR or macular edema varies among individuals. Several studies have estimated that the cumulative rate of progression from non-proliferative stages to vision-threatening stages ranges between 14 and 16% [2,3]. While the etiology remains poorly known, DR is clearly a complex multifactorial disease caused by a combination of genetic, environmental, and immunological factors [4,5,6,7,8]. Multiple immune mediators have been identified in eyes with PDR and diabetic macular edema, suggesting a pathogenetic role of the mediators.

However, there is no report to date which describes immune mediator concentrations in the ocular fluids of eyes with simple DR. Recently, two-color flow cytometry has been used for the simultaneous detection of many immune mediators using a very small volume of a sample such as aqueous humor [9]. The predominant advantage of cytometric bead array (CBA) technology lies in its ability to measure multiple parameters concurrently using a relatively small sample volume such as aqueous humor, making it faster and more cost-effective than ELISA technology. Previous studies on cytokines or chemokines associated with PDR or diabetic macular edema quantified a small number of cytokines or chemokines using the Enzyme-linked Immunosorbent Assay (ELISA) system [10,11]. Because single-assay measurements of immune mediators provide limited information, multiple immune mediators measured simultaneously must be analyzed to obtain a more comprehensive picture of DR.

The purpose of this study was to identify and quantify a wide spectrum of immune mediators including cytokines, chemokines, growth factors, and apoptosis-related molecules in aqueous humor samples collected from eyes with simple DR. Understanding how immune mediators are associated in the early stages of simple DR may shed light on the pathophysiology of DR progression and help to develop new biomarkers for early diagnosis.

## 2. Materials and Methods

### 2.1. Subjects

In this retrospective study, 15 patients (15 eyes) with simple DR were identified from the medical records between September 2011 and May 2022, and 22 patients (22 eyes) with cataract and no diabetic mellitus (DM) were included as disease controls. The 15 patients (11 males and 4 females) diagnosed with simple DR according to Davis classification were 65.8 ± 11.6 years of age; all had type 2 diabetes mellites (T2DM) with mean hemoglobin A_1C_ (HbA_1C_) of 7.5 ± 1.4 and mean duration of diabetes of 12.1 ± 7.5 years (Table 1). The 22 cataract patients (9 males and 13 females) with no DR based on Davis classification were aged 72.1 ± 8.8 years. All patients were Japanese adults. The absence of diabetic macular edema was defined as no retinal thickening at the macula based on clinical and OCT examinations. This study was reviewed and approved by the institutional review board of Tokyo Medical University. Informed consent was obtained from each participant, who were provided with explanations regarding the purpose and methods of the study for effective disease control.

### 2.2. Measurements

Undiluted aqueous humor samples (approximately 100 μL) were collected via an anterior chamber tap with a 25 needle from patients with simple DR during outpatient consultation, and from cataract patients with no DM before cataract surgery. All samples were stored at −80 °C until use. The CBA Flex immunoassay kit (BD Biosciences, San Jose, CA, USA) was used to determine the aqueous humor concentrations of 32 immune mediators comprising interleukins (IL)-1α, IL-1β, IL-2, IL-3, IL-4, IL-5, IL-6, IL-8, IL-9, IL-10, IL-12p70, IL-17A, and IL-21, interferon (IFN)-γ, interferon-γ-inducible protein (IP)-10, monocyte chemoattractant protein (MCP)-1, macrophage inflammatory protein (MIP)-1α, MIP-1β, regulated on activation, normal T-cell expressed and secreted (RANTES), monokine induced by interferon-γ (Mig), basic fibroblast growth factor (bFGF), Fas ligand, granzyme A, granzyme B, interferon-inducible T-cell alpha chemoattractant (ITAC), fractalkine, granulocyte macrophage colony-stimulating factor (GM-CSF), granulocyte colony-stimulating factor (G-CSF), vascular endothelial growth factor (VEGF), angiogenin, tumor necrosis factor (TNF)-α, and CD40 ligand. This method allows the simultaneous detection of many analytes with a very small volume of sample (100 µL), as described previously [12].

### 2.3. Analysis for the Interaction of Altered Immune Mediators

To elucidate the interaction network of immune mediators with significantly altered expression levels in simple DR, we utilized Metascape [13] (https://metascape.org/, accessed on 5 November 2023). The STRING database ver.12 [14] (https://string-db.org/, accessed on 5 November 2023) was used to visualize the protein–protein interactions among the immune modulators with significantly altered aqueous humor concentrations in simple DR compared to disease controls.

### 2.4. Statistical Analysis

Statistical analyses were performed using JMP version 10 (SAS, Cary, NC, USA) and the graphs were generated using GraphPad Prism 9 (ver. 9.5.1). Two-group comparisons of categorical variables were performed using Fisher’s exact test. Continuous variables were compared using Student’s *t*-test or a Mann–Whitney U test depending on the normality of the data distribution. Specifically, we employed the non-parametric Mann–Whitney U test to analyze immune mediator concentrations since the data were not normally distributed. Data in the text and table are presented as mean ± standard deviation or median with interquartile range in parenthesis. Immune mediator concentrations below the lowest limit of detection were treated as 0 pg/mL in statistical analyses. The significance level for all tests was 5%.

## 3. Results

The demographic data of the simple DR group and control group are shown in Table 1. Table 2 presents the immune mediator concentrations in the simple DR group and control group. Aqueous humor concentrations [mean (interquartile range)] of nine immune mediators comprising bFGF, CD40 ligand, fractalkine, G-CSF, IL-6, IL-8, MIP-1α, MIP-1β, and VEGF were significantly higher in simple DR than in controls (Table 2). On the other hand, Fas ligand concentration in aqueous humor was significantly lower in simple DR than in controls. The aqueous humor concentrations of some immune mediators including Fas ligand and IL-9 in all patients with simple DR, and CD40 ligand, G-CSF, IL-1α, IL-5, IL-7, IL-9, TNF-α, and ITAC in all controls, were below the lowest limits of detection in all samples.

Notably, the aqueous humor concentration of IL-8 in simple DR was significantly different compared to controls [13.47 (0–153) pg/mL versus 3 (0–15.99) pg/mL, *p* = 0.01] in this study. This aligns with previous reports, indicating a significant upregulation of aqueous humor IL-8 concentration in diabetic macular edema and PDR relative to controls [15,16]. Therefore, IL-8 upregulation may play a key role even in the very early stages of diabetic retinopathy.

**Figure 1:**
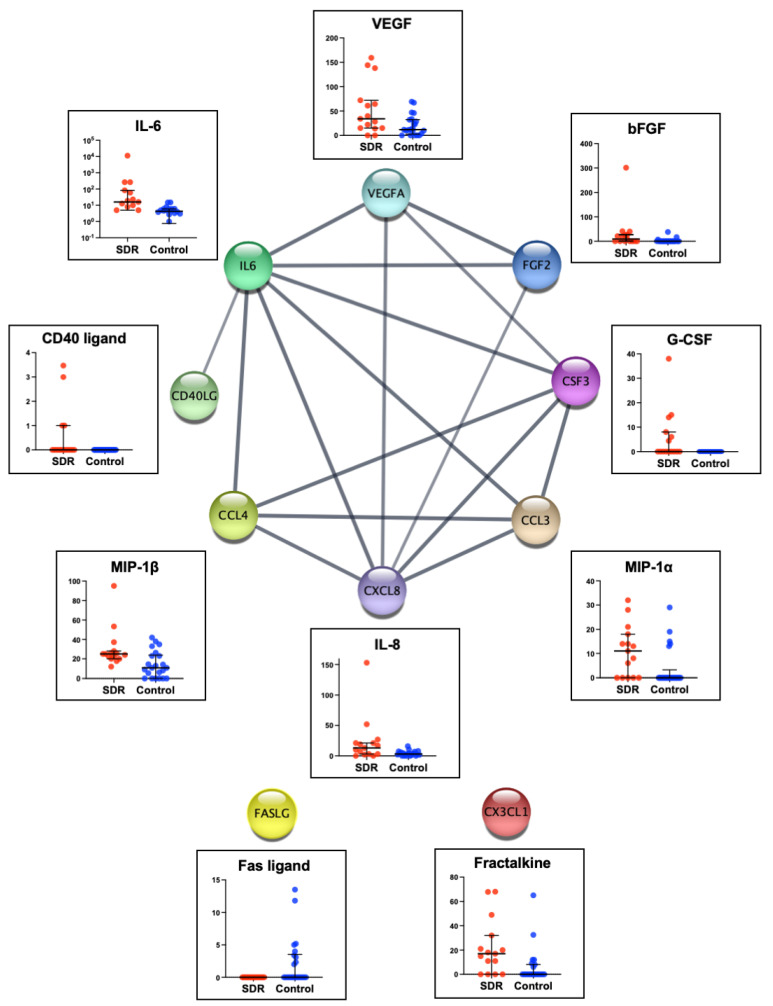
The graph generated from the STRING database represents protein–protein interactions among immune modulators that exhibited significant alterations in aqueous humor concentration in simple diabetic retinopathy. Each node, labeled by the cytokine name, is linked to an associated cytokine through a connection termed an “edge.” Next to each node, a bar graph shows the aqueous humor concentration from this study. Among the 10 immune mediators, eight cytokines established edges with multiple cytokines. Conversely, Fas ligand and fractalkine showed no interactions with others.

In addition, we investigated the relationship among immune mediators with altered aqueous humor concentrations in simple DR. Among the 10 cytokines with significantly altered aqueous humor concentrations, a protein–protein interaction analysis using the STRING database revealed that eight were closely interconnected, forming a complex interaction network (Figure 1). These results suggest that diabetic retinopathy progresses via the interaction of multiple pathways, rather than through a single altered pathway.

## 4. Discussion

The etiology of DR remains largely unknown. Exposure to hyperglycemia over an extended period is considered to promote changes that cause vascular endothelial impairment. The early histologic features of DR are capillary basement membrane thickening, leukostasis, a loss of endothelial cells, and a loss of pericytes [17,18]. Sustained capillary occlusion causes hypoxia that is further accelerated by the release of VEGF and other immune mediators [19,20], leading to the development of intraretinal microvascular abnormalities, providing alternative collateral routes for blood to travel from the arteries to the veins. In up to 20% of patients with diabetes, ischemia of the inner retina secondary to the closure of parts of the retinal capillary bed leads to neovascularization on the surface of the retina and optic disc, signaling the presence of PDR [21].

Sustained hyperglycemia is considered to be the major initiating factor of DR. However, detailed mechanisms causing retinal abnormality remain unclear. The disease involves the progression of retinovascular damage with stepwise clinical progression from mild stages to advanced proliferative changes. The rate of progression varies among patients and depends mainly on systemic factors such as blood glucose level, blood pressure control, and blood lipid profile, which constitute independent risk factors of DR development [22]. Our results suggest that alterations to the aqueous humor concentrations of immune mediators in patients with simple DR may reflect the early stages of disease progression.

DR tends to cause ocular inflammation. Although the precise mechanisms underlying such chronic inflammation are not yet known, macrophages and many immune mediators including angiogenic growth factors, cytokines, and chemokines are involved in the onset and progression of DR. In advanced stages of DR, such as PDR and diabetic macular edema, immune cells and mediators play important roles in disease progression [19,20,23,24,25]. Indeed, macrophages have been shown to play a pivotal role in PDR and diabetic macular edema development, by invading the retina [24,26,27]. High levels of VEGF, a potent proangiogenic factor, are expressed in the retinas of diabetic patients, resulting in marked increases in intraocular VEGF concentration correlating with the presence of soft exudates, intraretinal microvascular abnormality, venous bleeding, venous loops, and neovascularization [19,28]. Genetic polymorphisms in VEGF-A have also been associated with an increased risk of developing proliferative DR, further corroborating the critical role of this factor in DR [29]. Vitreous and aqueous concentrations of VEGF and IL-6 may serve as biomarkers of disease progression, because they correlate with the severity of macular thickness alteration in diabetic macular edema [30]. The CD40 ligand is a member of the TNF receptor superfamily and is expressed on inflammatory cells, primarily activated CD4+ T cells, as a receptor for CD40. The CD40-CD40 ligand pathway plays a significant role in both autoimmunity and adaptive immunity [31,32], Studies have suggested that this pathway also contributes to the pathophysiology of diabetes mellitus [33,34]. Lamine et al. reported that circulating soluble CD40 ligand concentration in serum was associated with the severity of diabetic retinopathy in patients with T2DM [35]. In this study, the CD40 ligand concentration in aqueous humor was elevated in early-stage DR. Combined with previous research, this finding suggests the potential involvement of the CD40 ligand in diabetic retinopathy pathogenesis from the onset. Notably, the Fas ligand, an apoptosis-inducing ligand, was the sole cytokine showing a decrease in simple DR compared to controls. Previous in vitro studies demonstrated the suppression of Fas ligand expression by VEGF [36], which aligns with our observation of reduced Fas ligand concentrations in the aqueous humor of patients with simple DR.

Among the nine immune mediators that were upregulated in simple DR in this study, fractalkine, also known as chemokine CX3C Receptor 1, is particularly interesting for its dual roles as a chemotaxin and an activator of retinal microglia and infiltrating macrophages, as well as being a physiologically active compound that potently increases vascular permeability and promotes angiogenesis [37,38,39]. In addition, a previous study in mice suggested that microglia modulate neurovascular function in the retina, and dysfunction of the microglial–vascular function may potentially cause early vascular compromise, leading to the development of DR [40]. Therefore, the altered cytokines identified in this study may reflect the responses of various cells, including vascular endothelial cells, inflammatory cells, and retinal microglia, due to diabetic vascular abnormality.

Of the ten cytokines that showed significant alterations in simple DR in our study, nine immune mediators form a tightly interwoven and complex interaction network. This finding suggests that many immune mediators act synergistically, interacting to trigger the development of DR. Further research is warranted to uncover the functional roles of these 10 cytokines in the initiation and progression of early-stage DR.

This study has several limitations. First, because retrospectively collected samples were used, the time from HbA1c measurement or disease onset to aqueous humor collection varied among samples. This may suggest a variable degree of disease progression across the samples. Second, the single-center case–control study design may give rise to potential sampling or clinical biases, including collection bias, in specific periods related to geographic location, ethnicity, age, and sex distribution. Third, selection bias when using cataract patients instead of healthy controls is included as a limitation. Notably, the simple DR group in this study consisted of predominantly males, which is a potential bias. In addition, our sample size was small to detect the generalized alternation with simple DR. To address these concerns, future prospective multi-center studies on a global scale would be needed to validate the present findings.

## 5. Conclusions

The aqueous humor concentrations of 10 immune mediators comprising bFGF, CD40 ligand, Fas ligand, fractalkine, G-CSF, IL-6, IL-8, MIP-1α, MIP-1β, and VEGF were altered in patients with simple DR. Although this is the first study of the aqueous humor immune mediator profile in simple DR, our results are mostly in agreement with previous studies showing that various immune mediators associated with inflammation and angiogenesis are related to the development of DR. Combinations of these immune mediators may also be useful biomarkers for the early detection of DR and the prediction of prognosis. Future large-scale studies are needed to evaluate whether altered aqueous humor concentrations of these cytokines or their interactions are associated with an increased risk of progression to advanced stages of DR.

## Figures and Tables

**Table 1:** Demographic and clinical data of patients with simple diabetic retinopathy (DR), and disease controls.

		Subject	Control
Number of eyes		15	22
Number of cases		15	22
Sex	Male	11	9
	Female	4	13
Age (years)		65.8 ± 11.6	72.1 ± 8.8
HbA1c (%)		7.5 ± 1.42	―

**Table 2:** Immune mediator levels in aqueous humor of patients with simple diabetic retinopathy.

	Simple DR		Controls		
	(*n* = 15)		(*n* = 22)		
Name	Median	Range	Median	Range	*p* Values
Angiogenin (pg/mL)	4468.9	1495–58426	5257	943–9489	0.614
bFGF (pg/mL)	9	0–301	0	0–38	0.026
CD40 ligand (pg/mL)	0	0–3.47	0	0–0	0.042
Fas ligand (pg/mL)	0	0–0	0	0–13.5	0.036
Fractalkine (pg/mL)	16.5	0–68	0	0–65	0.007
G-CSF (pg/mL)	0	0–38	0	0–0	0.042
GM-CSF (pg/mL)	0	0–4.86	0	0–4.8	0.819
Granzyme A (pg/mL)	0	0–6.51	0	0–13.5	0.262
Granzyme B (pg/mL)	0	0–133.44	0	0–13.5	0.725
IFN-γ (pg/mL)	0.5	0–3.93	0	0–6.64	0.939
IL-1α (pg/mL)	0	0–7.62	0	0–0	0.748
IL-2 (pg/mL)	0	0–76.6	0	0–17.8	0.593
IL-3 (pg/mL)	0	0–8.04	0	0–4.8	0.531
IL-4 (pg/mL)	0	0–9.23	0	0–2	0.915
IL-5 (pg/mL)	0	0–3.43	0	0–0	0.092
IL-6 (pg/mL)	15.9	0–11230	4.3	0–15.03	<0.001
IL-7 (pg/mL)	0	0–112	0	0–0	0.181
IL-8 (pg/mL)	13.47	0–153	3	0–15.99	0.01
IL-9 (pg/mL)	0	0–0	0	0–0	1
IL-10 (pg/mL)	0	0–18.6	0	0–3.7	0.135
IL-12p70 (pg/mL)	0	0–96.8	0	0–13.5	0.075
IL-17A (pg/mL)	0	0–26.2	0	0–4.68	0.065
IL-21 (pg/mL)	0	0–181	0	0–17	0.829
IP-10 (pg/mL)	89.2	0–873	84.5	0–417	0.843
MCP-1 (pg/mL)	494.5	77–11859	327.8	72–780	0.152
Mig (pg/mL)	32	8–187	18.4	0–174	0.237
MIP-1α (pg/mL)	10.7	0–32.4	0	0–29	0.039
MIP-1β (pg/mL)	24.7	12.3–94.9	10.8	0–42	0.005
RANTES (pg/mL)	0.5	0–31.5	0	0–3.8	0.225
TNF-α (pg/mL)	0	0–0.84	0	0–0	0.748
VEGF (pg/mL)	34	0–159	11.9	0–69.3	0.033
ITAC (pg/mL)	0	0–17	0	0–0	0.511

Disease controls were patients with cataracts and no diabetic retinopathy. Immune mediator levels are expressed as median with interquartile range in parenthesis. bFGF, basic fibroblast growth factor; G-CSF, granulocyte-colony stimulating factor; GM-CSF, granulocyte macrophage-colony stimulating factor; IFN, interferon; IL, interleukin; IP-10, interferon gamma-induced protein 10 kDa; ITAC, interferon-inducible T-cell alpha chemoattractant; MCP, monocyte chemoattractant protein; Mig, monokine induced by interferon γ; MIP, macrophage inflammatory protein; RANTES, regulated upon activation, normal T expressed, and presumably secreted; TNF, tumor necrosis factor; VEGF, vascular endothelial growth factor.

## Data Availability

The data presented in this study are available on request from the corresponding author.

## Abbreviations

DR: Diabetic retinopathy; PDR: proliferative diabetic retinopathy; SD: standard deviation.
